# Smartphone-Based Ecological Momentary Assessment for Collecting Pain and Function Data for Those with Low Back Pain

**DOI:** 10.3390/s22187095

**Published:** 2022-09-19

**Authors:** Ekjyot Kaur, Pari Delir Haghighi, Flavia M. Cicuttini, Donna M. Urquhart

**Affiliations:** 1 Department of Human-Centred Computing, Faculty of Information Technology, Monash University, Clayton, Melbourne, VIC 3800, Australia; 2 Department of Epidemiology and Preventive Medicine, School of Public Health and Preventive Medicine, Alfred Hospital, Monash University, Clayton, Melbourne, VIC 3800, Australia

**Keywords:** ecological momentary assessment, model of technology appropriation, mobile health monitoring, smartphone-based data collection, low back pain

## Abstract

Smartphone-based ecological momentary assessment (EMA) methods are widely used for data collection and monitoring in healthcare but their uptake clinically has been limited. Low back pain, a condition with limited effective treatments, has the potential to benefit from EMA. This study aimed to (i) determine the feasibility of collecting pain and function data using smartphone-based EMA, (ii) examine pain data collected using EMA compared to traditional methods, (iii) characterize individuals’ progress in relation to pain and function, and (iv) investigate the appropriation of the method. Our results showed that an individual’s ‘pain intensity index’ provided a measure of the burden of their low back pain, which differed from but complemented traditional ‘change in pain intensity’ measures. We found significant variations in the pain and function over the course of an individual’s back pain that was not captured by the cohort’s mean scores, the approach currently used as the gold standard in clinical trials. The EMA method was highly acceptable to the participants, and the Model of Technology Appropriation provided information on technology adoption. This study highlights the potential of the smartphone-based EMA method for enhancing the collection of outcome data and providing a personalized approach to the management of low back pain.

## 1. Introduction

Recent advancements in mobile technologies, especially smartphones, have made them useful tools for collecting health-related data in real time and on a frequent basis [1,2]. Smartphone-based ecological momentary assessment (EMA) is an effective data collection method that uses smartphones for the repeated sampling of an individual’s symptoms and activities in real time in their natural environment, which in turn, maximizes the ecological validity [3]. The EMA combines both ecological and momentary aspects. The ecological aspect refers to data that are collected in the individual’s natural environment, and the momentary aspect refers to the use of shorter intervals for collecting data [4]. EMA has been successfully used in a wide range of health conditions such as fibromyalgia [5], spinal cord injury [6], and low back pain [7]. Smartphone-based EMA methods present an opportunity to collect health-related data, such as pain, as it happens, which allows for the collection of sensory data about functional activity from the device’s built-in sensors over the course of an individual’s daily activities. However, the adoption of smartphone-based EMA methods for data collection has been limited to clinical trials [8,9]. There is an urgent need for a better understanding of the feasibility and full potential of these methods and how they could be adopted to complement and improve existing practices.

Low back pain is a major public health issue in which EMA could play an important role. Low back pain is not only highly prevalent but the leading cause of disability globally [10]. Despite this, there is a lack of effective treatments for the condition [11]. There is also a major concern as high-quality, randomized controlled trials involving patients with low back pain are continually finding minor benefits of potential treatments that are not clinically important, leading to conclusions that these therapies are ineffective [11]. Clinical trials involving patients with low back pain typically assess the benefits of interventions by assessing the outcomes of pain intensity and disability (as a proxy for function) using self-reporting questionnaires at two discrete time points at sparse intervals (i.e., usually 3 to 6 months apart). Although this is currently the gold standard approach to the measurement of outcomes in low back pain research, it is possible that the use of this static, two-time-point approach is a major contributing factor to potential treatments being found to be ineffective. This method may mean that the burden of low back pain in the individual and the efficacy of treatment approaches are missed. In addition, low back pain is well-recognized as a complex, heterogeneous condition, in which different patient subgroups differ in their pathophysiology and treatment approaches. Currently, the measurement of outcomes is focused on a ‘one-size-fits-all’ approach, where the effectiveness of the interventions is based on analyzing the difference between the mean change scores for the intervention and control groups. This method does not take into account that there will be differences in an individual’s pain experiences and function over the course of their low back pain.

This study aims to address this research gap and presents the implementation and evaluation of a smartphone-based EMA method for collecting pain and function data in individuals with low back pain. The aims of this study were to:

1. Determine whether the outcome measures of pain intensity and function can be collected using smartphone-based EMA methods in real time, on a frequent basis, and in the user’s natural environment to characterize an individual’s pain experience over time;
2. Investigate whether the pain intensity measures collected using EMA methods are similar or different from traditional measures of collection;
3. Examine the relationship between the measures of pain intensity and function obtained with smartphone EMA methods;
4. Determine the appropriation and satisfaction levels of individuals that used the smartphone-based EMA methods using the MTA and identify the key attractors, appropriation and non-appropriation criteria, and reinforcers to optimize the interface, functionality, and patient satisfaction.

We hypothesize that smartphone-based EMA is a feasible method for the collection of pain and function data in individuals with low back pain. The data collected using the EMA method differs from traditional methods and highlights that there is significant variation between individuals in their pain experiences and the relationship between pain and function. Moreover, the findings show that there is high participant satisfaction with the approach, and key factors that influence EMA adoption are identified.

The rest of the paper is organized as follows. Section 2 presents the user study methodology that involved the development of the mobile app for data collection. Section 3 discusses the user study design, sample, and procedures for collecting and analyzing the data. Section 4 describes the results of the implementation of the EMA method for collecting the pain and function data and describes the findings through the lens of the MTA theory. Section 5 discusses the findings and limitations, and Section 6 concludes the paper.

## 2. The User Study Methodology

### Smartphone-Based EMA Data Collection App Development

**Figure 1:**
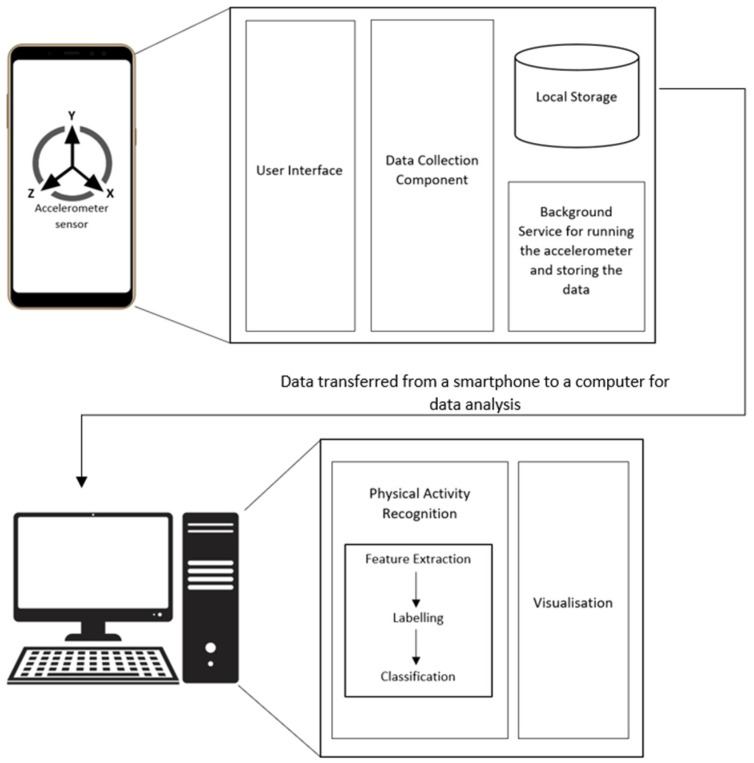
The architecture of the smartphone-based EMA data collection.

Figure 1 shows the architecture of the smartphone-based EMA data collection system. The main component of this system was a mobile app that was developed in Android and installed on Nokia TA-1079 phones. The mobile app was designed and developed to collect pain intensity and function data (walking and sitting). Domain experts were actively involved throughout the design, prototype development, and implementation processes of the app and provided valuable feedback for its improvement.

**Figure 2:**
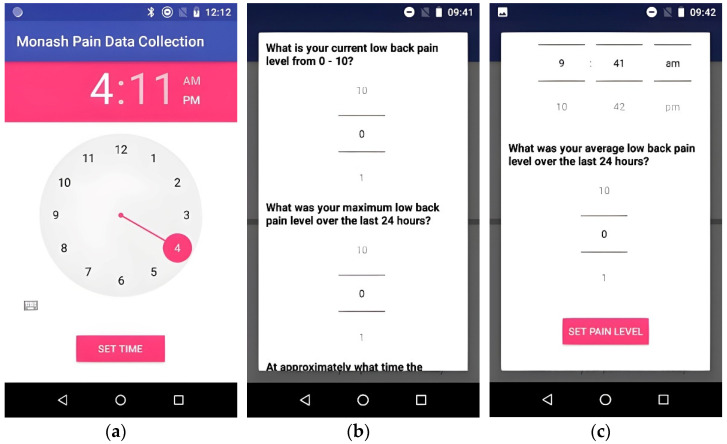
Mobile application screenshots: (**a**) to set a fixed daily time for pain data entry, (**b**) to enter the current and the maximum pain intensity over the last 24 h, and (**c**) to enter the average pain intensity over the last 24 h.

**User Interface**—The mobile app had a simple user interface that allowed participants to set a fixed time to enter their current pain intensity, maximum pain intensity, and average pain intensity at the beginning of the study. The pain intensity ranged from 0 to 10, where 0 indicated no pain and 10 indicated the worst pain imaginable. The participants could also set an alarm as a reminder. The reminder feature was used to ensure the pain data was entered at a fixed time daily. Figure 2 shows the screenshots of these interfaces.

**Data Collection Component**—This component was responsible for collecting the accelerometer data as x, y, and z values with associated timestamps from the smartphone’s built-in accelerometer that was used to determine function (sitting or walking).

**Background Service**—The app included an Android service that was running in the background to enable the data collection component to continuously collect the accelerometer data with no involvement from the participants. The background service was also responsible for automatically storing the collected data daily in the smartphone’s local storage. It was vital that this component worked without any interruptions over the entire study period.

**Physical Activity Recognition**—At the end of the study, the function data stored on the smartphones were transferred to a laptop where the activity recognition was performed. The physical activity recognition included feature extraction and classification. Feature extraction was responsible for the selection of features in the classification. The collection of accelerometer data generated time-series instances of physical activity. The commonly used features were frequency–domain and time–domain features. The classification algorithm generated the classification results based on the data input instances and selected features for sitting and walking.

**Visualization**—This component was used for creating different graphs to examine the relationship between the measures of pain intensity and function.

## 3. User Study

### 3.1. Study Design and Protocol

The EMA protocol created for this study is presented in Table 1. The study was conducted over seven months, with a study period of two weeks for collecting the data from each participant. The data collection instruments for the study were (i) the developed mobile app, which collected data on pain and function on a daily basis from each participant (Figure 2), and (ii) the paper-based questionnaires for pain and function that are currently used in low back pain clinical trials and were completed by participants at the start and end of the 2-week period.

### 3.2. Study

Participants were included in the study if they were adults aged 18 years or above, presented with a primary complaint of low back pain that was either sub-acute (3–12 weeks) or chronic (>12 weeks), and had an average pain intensity of 3 or more on a 0–10 visual analogue scale. The exclusion criteria included any spinal fracture, trauma to the spine, inflammatory back pain, low back surgery or other invasive procedure within the previous 3 months, current pregnancy, or inability to provide informed consent. We recruited individuals from a database of community-based individuals with low back pain. These individuals had expressed interest in being part of a clinical trial examining a new intervention for low back pain but some were ineligible or chose not to be involved after receiving further information about the study.

### 3.3. Procedure

The study was first discussed with the participants by phone and detailed information was provided about the study. If the individual indicated they were interested in taking part in the study, verbal consent was obtained to undertake screening, the screening protocol was conducted, and participants’ responses were recorded.

If the individual met the eligibility criteria and wished to proceed with the study participation, an appointment was made to obtain informed consent and collect baseline data. The informed consent procedure was performed according to Good Clinical Research Practice (which includes explaining the collection and storage of data) [12]. At the baseline visit, validated outcome measure questionnaires were administered that are currently used in clinical trials of patients with low back pain. These included the visual analogue scale (VAS) [13], Low Back Pain Rating Scale [14], Roland Morris Disability Questionnaire [15], and International Physical Activity Questionnaire [16]. The outcome measure questionnaires were administered using self-reporting, paper-based forms and were completed at a single time at two discrete intervals, i.e., baseline and 2 weeks. The research staff showed the participants how to use the mobile data collection app and its main functionality. This involved the participants unlocking the smartphone, tapping the ‘Start’ button on the mobile application, and recording their current pain intensity, maximum pain intensity, and average pain intensity scores on a scale of 0–10, as per the Low Back Pain Rating Scale.

Participants were asked to carry the smartphone for at least 10 h per day for 2 weeks to collect the pain and function data, including walking and sitting. In addition, the participants were requested to nominate their preference for the time of day when they would like to enter their pain intensity data during the study. The smartphone reminded the participant to enter their pain intensity manually into the smartphone at the nominated time each day.

At the end of week 1, the participants were called and interviewed regarding their initial experience with the mobile data collection application. This included the System Usability Scale (SUS). The usability questionnaire was divided into three sections: (i) demographics, (ii) SUS’s 10-item usability scale, and (iii) nine open-ended questions. At the end of the study (week 2), the baseline paper-based and usability questionnaires were repeated to obtain the outcome data at follow-up and assess the mobile pain data collection acceptance in real time.

The usability evaluation was used to assess the usability and user interface of the mobile app for mobile pain data collection and understand user satisfaction and acceptance with regard to carrying the phone around their waist over the course of the study. The nine open-ended questions in the usability questionnaire aimed to seek feedback about these aspects, as well as other features of the mobile app, such as the reminder feature. The responses to the open-ended questions were qualitatively analyzed using the MTA to better understand the adoption of the mobile data collection app in the user’s daily routine and the key factors that influenced it.

### 3.4. Data Analysis

To analyze the pain intensity data, the ‘change in pain intensity’ and ‘pain intensity index’ scores were calculated. The ‘change in pain intensity’ was calculated by subtracting the pain intensity at baseline from the pain intensity at the 2-week follow-up. The ‘pain intensity index’ was calculated by summing the daily pain scores and dividing this by the total number of days to provide a measure of the burden of pain. Missing data were imputed by calculating the average score of the previous day and the next day, or if this was not possible, the average score of the two days prior to or after the missing value. We found that 2.5% (21/855) of the data points were missing.

To analyze the data related to function, a ‘walking index’ was calculated by summing the total daily walking minutes over the 2 weeks and dividing this by the total number of days. Similarly, the ‘sitting index’ was calculated by adding the total daily sitting minutes over the 2 weeks divided by the total number of days. The walking and sitting indices allowed us to explore the relationship between pain intensity and function data.

To examine the satisfaction and appropriation of the EMA method, interviews were transcribed manually, and thematic analysis was applied. The thematic analysis consists of six main steps: understanding the data, generating initial codes, building themes, reviewing themes, defining and organizing themes, and interpreting and writing them up [17]. NVivo software was used to help with the coding step. Through an iterative process, the themes that emerged from the data were refined based on the feedback from the domain experts involved in the study.

## 4. Results

### 4.1. Participant Characteristics

A total of 19 participants were included in the study. The baseline characteristics of the cohort are presented in Table 2. The mean (SD) age and body mass index (BMI) were 47.6 (12.3) years and 27.9 (4.61) kg/m^2^, respectively, and nine participants (47.4%) were females. Of the study cohort, 8 (42.1%) participants had a bachelor’s or higher degree, 14 (73.3%) participants were employed full-time, and 15 (78.9%) performed office or professional work.

### 4.2. Average Measures of Pain Intensity in the Cohort

**Figure 3:**
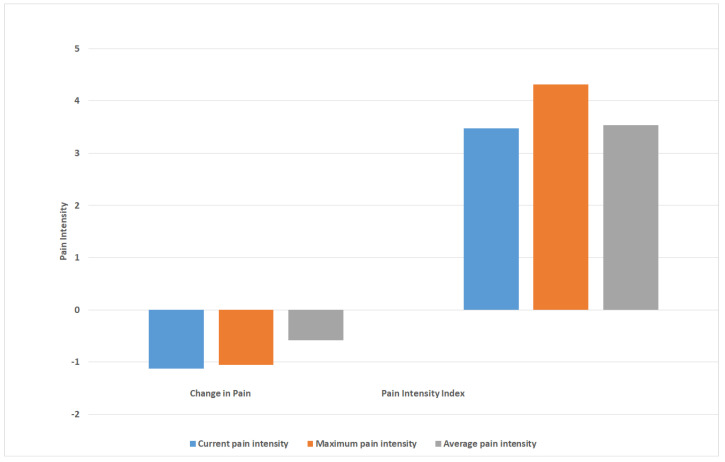
Pain intensity measures for the cohort over 2 weeks, including measures of ‘change in pain’ (difference in pain scores between follow-up and baseline) and ‘pain intensity index’ (average of pain intensity over 2 weeks).

Figure 3 presents the pain measures of (i) ‘change in pain intensity’ (difference between follow-up and baseline) and (ii) ‘pain intensity index’ (average of pain intensity over the 2 weeks) for the cohort over 2 weeks. The mean (SD) ‘change in pain intensity’ scores for the cohort for the current, maximum, and average pain were −1.1 (2.3), −1.1 (2.5), and −0.6 (2.0), respectively. The mean (SD) ‘pain intensity index’ scores for the current, maximum, and average pain were 3.5 (1.8), 4.3 (1.8), and 3.5 (1.8). The mean ‘change in pain scores’ indicated that the cohort improved by 0.5–1 on a pain intensity scale of 0–10, whereas the mean ‘pain intensity index’ scores showed an overall pain intensity level of 3–4 for the group over the course of the study.

### 4.3. Variations in Measures of Pain Intensity in Individual Participants

**Figure 4:**
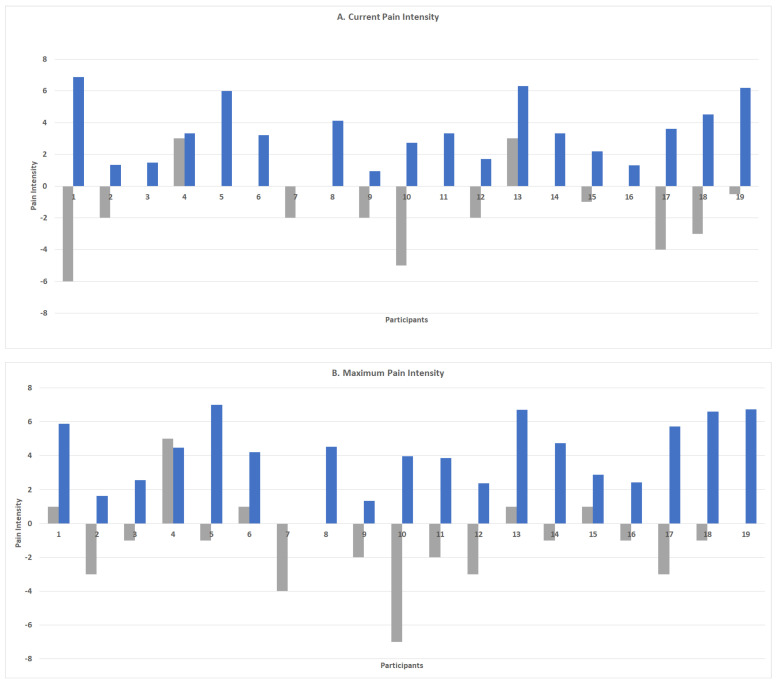

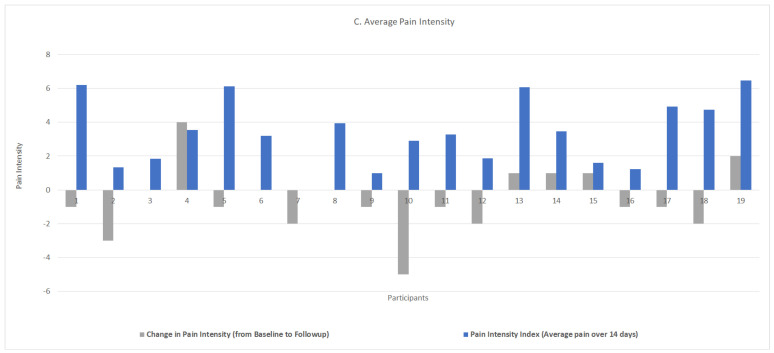
Measures of ‘change in pain intensity’ (difference between follow-up and baseline scores) and ‘pain intensity index’ (average of pain intensity scores over 2 weeks) for each individual in the cohort.

Figure 4 presents the mean ‘change in pain intensity’ and ‘pain intensity index’ scores for each of the 19 participants. The data show that although individuals could have a ‘change in pain intensity’ score that indicated an improvement in pain (negative score), they could also have a ‘pain intensity index’ that showed overall moderate-to-high levels of pain intensity for the study period. For example, although participant 1 was found to have a mean ‘change in pain intensity’ score of 6, indicating an improvement of six increments on the 0–10 mm pain scale between baseline and follow-up, their mean ‘pain intensity index’ score was 6.9, indicating that overall, they experienced moderate-to-high pain over the 2 weeks. Moreover, the data show that two individuals could both have ‘change in pain intensity’ scores indicating an improvement in pain, but differed in their ‘pain intensity index’, with one score indicating a low level of pain experienced over the course of the study and the other a moderate level of pain (e.g., Figure 4B, participants 12 and 17).

### 4.4. Variability in Measures of Pain Intensity and Function (Walking and Sitting) across Individuals

**Figure 5:**
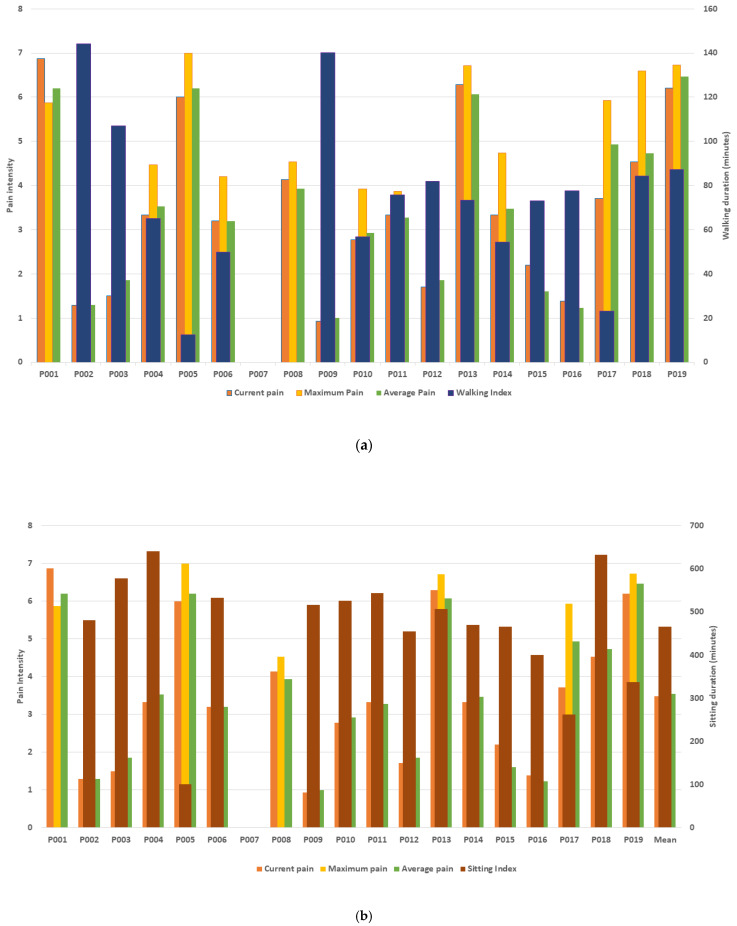
The mean current, maximum, and average pain intensity and mean daily walking (**a**) and sitting (**b**) durations (minutes) for each of the 19 participants.

The mean (SD) walking and sitting durations for the cohort were 75.4 (40.0) and 465.4 (133.6) minutes per day. Figure 5 presents the mean current, maximum, and average pain intensity scores for each participant in conjunction with their mean walking (a) and sitting (b) duration scores over the 2 weeks.

The data show that participants with high pain scores had both short and long mean walking durations over the study period. For example, participant 19 with pain levels of 6–7 walked an average of 87 min per day, whereas participant 5 with similar pain levels walked an average of 12 min. Similarly, participants with lower pain intensity scores performed different daily amounts of walking. Although participant 9 with pain levels of 1 walked an average of 140 min per day, participant 15 with similar pain levels walked an average of 73 min daily.

Participants with high pain levels also recorded sitting for different mean durations of time (Figure 5b). For example, participant 5 with pain levels of 6–7 had an average sitting duration of 100 min per day, whereas participant 15 with similar pain levels sat for an average of 465 min daily. Moreover, participants 16 and 9 with low levels of pain of 1–2 recorded sitting durations of 400 and >500 min, respectively.

### 4.5. Usability Testing

We assessed the usability of the mobile data collection app using a usability questionnaire. The usability questionnaire included 10 items of the SUS on a 5-point Likert scale [18,19]. The scores were allocated and calculated to obtain the overall mean score according to Brook’s guidelines [18]. The SUS mean score was 84.1. A SUS score above 71.4 and below 85.5 can be considered “good” [20]. The usability questionnaire also included open-ended questions that were transcribed and qualitatively analyzed through the lens of the Model of Technology Appropriation (MTA) [21], which are discussed in the following sections.

### 4.6. Satisfaction and Appropriation of the Proposed EMA Method

The study shows that the majority of participants (15 out of 19) were satisfied with the use of the smartphone-based EMA pain data collection app. A set of factors were identified that patients found to be useful (attractors and appropriation criteria), as well as disappropriation criteria that could have contributed to rejecting or discontinuing the use of the smartphone-based EMA pain data collection app. The qualitative findings also included considerations for the usability and design of the user interface of the mobile pain data collection app.

#### 4.6.1. Level 1 Attractors

**Fashion/Style:** The fashion and style aspects were evaluated based on the phone size and weight and the pouch given to participants for carrying the mobile device. One participant described the pouch as:

*“light-weight and better than holding [the smartphone] in the purse”.*

**Features**: The ability to collect function data automatically and provide reminders to the participant to enter their pain intensity scores was a new concept to some participants who had no or limited experience with mobile applications. The reason for using reminders was to collect the data intensity at a fixed time every day to ensure the credibility and accuracy of the data and reduce the potential for bias, where people only respond when they have pain. One of the participants mentioned that:

*“Automatic reminders were good. They reminded me to fill in my pain levels at the same time every day”.*

Another participant reported that:

*“[Reminder] was the only way I would have remembered to record my pain level at the same time”.*

#### 4.6.2. Level 2—Appropriation Criteria

Level 2 of the MTA evaluates the use of the mobile pain data collection method and explains the appropriation and non-appropriation criteria. The appropriation criteria included ease of use, intuitive user interface, and simplicity of the instruction manual.

**Ease of Use:** Participants acknowledged that the mobile application used to collect the pain intensity and function data was easy to use. One of the participants compared the use of the mobile pain data collection method with the paper-based questionnaires and said that

*“[smartphones were] much easier to enter pain levels rather than having to use a paper format”.*

**Intuitive User Interface Design:** An intuitive user interface design aims to help the user easily understand the design and use it. It reduces the user’s cognitive load and the need to memorize any information [22]. In other words, an intuitive interface is almost self-explanatory and uses familiar elements that help participants easily learn and interact with the mobile device [23]. One of the participants commented that

*“It [user interface of the mobile app] was very clear and clean”.*

**Simple Instruction Manual**: The participants gave positive feedback regarding the simplicity of the manual. A participant made the following comment:

*“It’s [instruction manual] intuitive and simple”.*

The positive feedback confirmed that the instruction manuals should include relevant and useful pictures and easy-to-understand instructions to explain the full functionality of the mobile pain data collection app.

#### 4.6.3. Level 2—Non-Appropriation Criteria

**Snoozing the Alarm:** In this user study, participants were required to enter their pain intensity manually once a day at their suggested time. Sometimes, participants were busy at their specified time and did not know how to delay or snooze the alarm on the smartphone. The option for snoozing the alarm was provided to the participants; however, there were a few who did not realize how to use the snooze feature. One of the participants commented:

*“Could not delay alarm. If it went off, you could not choose to delay for an hour”.*

The feedback shows that whereas the reminders were helpful, they could be distracting and annoying. It is important that the design of alarms consider providing the user with easy options for cancelling or delaying the alarm.

**User Interface Elements:** Although participants reported positive feedback about the app’s interface, there were a few who reported that the user interface element used for entering the pain level, which was designed as a scrollbar, was not effective. Participants commented that:

*“I didn’t like scrolling through the pain questions for setting my pain levels”*

**Burden of Carrying Phone:** Although participants were required to carry the secondary phone with them in a pouch on their waist, there were a few who found this to be a burden. One of the participants commented that:

*“did not like [carrying the phone]. Had to disguise it underneath clothes”.*

Using a secondary phone has the benefit of it being used only for data collection purposes but it could be a burden for some users to carry two phones. It is worthwhile exploring the use of a user’s own mobile phone for data collection in future studies.

#### 4.6.4. Level 3—Reinforcers

Level 3 of the MTA refers to ‘technology in use’ that captures the ongoing use of the mobile pain data collection method. In this level, the mobile pain data collection method can be appropriated and integrated into participants’ daily routines. In this user study, the duration set for collecting the pain data (pain intensity and function) was two weeks. There were three reinforcers identified at the end of the study, i.e., the need for progress reports, the need for reports on the relationship between pain intensity and function, and a simple user interface.

**Need for Progress Charts:** The mobile application was used only for collecting pain data from participants and did not provide any progress summaries or progress reports on how they were doing. One of the participants commented on the lack of progress charts by saying that

*“The mobile application lacked a dashboard to show history”*

**Reports on Pain Intensity and Functional Activities:** The pain intensity and function data collected on the phone were used for investigating the relationship between the measures of pain intensity and function at the end of the study. These tests were not performed in real time and the results were not shared with the participants. One of the participants, interested in understanding their pain levels and function, advised that:

*“it would be good to have an ongoing visual representation of the relationship between activity and pain level!”*

**Simple User Interface:** All participants mentioned that the design of the mobile application was simple and easy to use. The simple user interface allowed the user to perform better and avoid any unnecessary complexities. One participant supported this by commenting:

*“The simple design of the mobile application kept me going for entering my pain levels”*

A mobile application should be designed in such a way that the user should focus on completing the task with minimum effort rather than understanding the design of the application.

**Figure 6:**
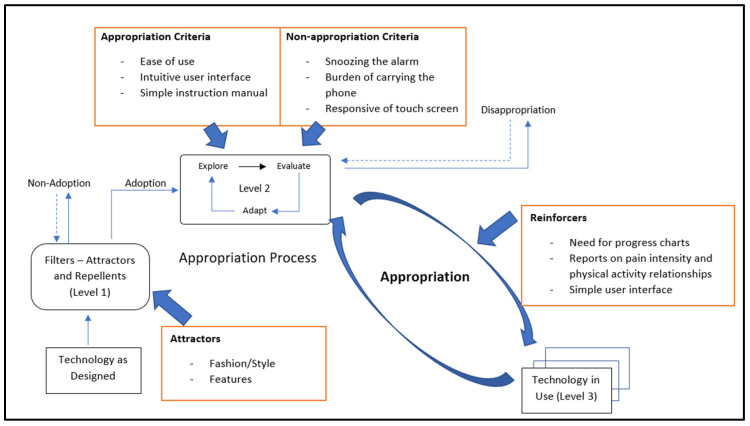
Results of the user study based on the Model of Technology Appropriation.

Figure 6 summarizes the factors that were identified through the qualitative data analysis using the MTA theoretical framework. These factors illustrate their relevance in the process of appropriation of the smartphone-based EMA method. There are three decision points in the MTA including the attractors, appropriation and disappropriation criteria, and reinforcers. As Figure 6 shows, the MTA framework was used to interpret the results under these constructs to provide us with an understanding of the adoption of the mobile data collection method among the participants. Overall, participants’ responses to the use of this method indicated the success of this study, where all the participants used the app for the duration of the study and there were no withdrawals.

## 5. Discussion

This user study showed that a smartphone-based EMA method involving the collection of data in real time, on a frequent basis, and in an individual’s natural environment can be used to collect pain and function data for individuals with low back pain. We found that calculating a ‘pain intensity index’ from the EMA data provided important information about the burden of pain over the course of the study and complemented traditional outcome measures that report a ‘change in pain intensity’. The results also highlighted the considerable individual variations in the measures of pain intensity and function, as well as the inter-relationships of these measures, indicating the need to consider a personalized, multidimensional approach to the assessment of outcomes. Participants were highly satisfied with the EMA approach, and the Model of Technology Appropriation (MTA) identified the key attractors, appropriation and non-appropriation criteria, and reinforcers for optimizing the interface, functionality, and patient satisfaction. This study highlights the potential of smartphone-based EMA methods in enhancing the collection of primary outcome data and providing a personalized approach to the management of low back pain.

We found that collecting pain data using the EMA method and calculating a ‘pain intensity index’ provided important information about the burden of low back pain over the study period. The ‘pain intensity index’, a summary measure of the daily pain values collected in real time over the study period, differed from the current outcome measures of the ‘pain intensity change score’, which indicates the change in pain intensity between two time points that are 3–12 months apart. For example, in the current study, although the ‘pain intensity change score’ showed that the cohort had experienced a reduction in pain of one increment on the 0–10 pain intensity scale, the ‘pain intensity index’ showed that the burden of pain for the cohort was at a moderate pain level of 4 over the course of the study. Similarly, at an individual level, although a participant reported a reduction in pain of 6, their average ‘pain intensity index’ score over the course of the study was 6, indicating a significant pain burden. This finding is novel. Research using smartphone-based EMA methodology in healthcare, and specifically in clinical trials of chronic pain, is still in its infancy. There is a lack of efficacious treatments for low back pain and high-quality clinical trials are consistently finding potential treatments to be ineffective. Given the insensitivity of the current approaches for assessing outcomes are likely to be contributing to this, there is an urgent need to improve our assessment of the primary outcomes of pain and function in clinical trials. The smartphone-based EMA methodology developed and implemented in this study shows exciting potential and provides insight into how we may further develop outcome methodologies to capture the pain experience in those with low back pain.

The study provides evidence of considerable individual variations in the outcome measures of both pain intensity and function within a low back pain cohort. We found that two individuals could record the same ‘change in pain’ score indicating an improvement in pain (i.e., 3 on a 0–10 scale) but differ in their ‘pain intensity indices’, with one individual experiencing a low burden of pain (score of 2) and the other a moderate-to-high burden (score of 6). Moreover, our data showed that participants with similar ‘pain intensity change scores’ (e.g., 6 on a 0–10 pain scale) could have large differences in their functional activities, including a difference of 75 min in their daily walking duration. These findings are important for two reasons. Firstly, they indicate the need to consider a personalized approach to characterizing and monitoring the progress of individuals with low back pain. It is evident from this study that focusing only on change scores at a cohort level means that the progress and pain experiences of the individual are missed. Instead, we need to consider an approach for characterizing and monitoring progress at an individual level over the course of low back pain. This is particularly important given that low back pain is considered a complex, heterogeneous condition and it is well accepted that treatments need to be tailored to individuals and specific patient subgroups. Secondly, these findings highlight the need to consider a combination of outcome measurements of pain and function for low back pain. It is clear that high levels of pain do not mean that an individual’s activity levels will be low, and conversely, low levels of pain are not necessarily associated with high levels of activity. The combination of pain and activity is important, provides a holistic approach, and needs to be captured in order to optimize patient results.

This user study was successful, with all participants using the smartphone EMA method to record their pain and functional activities for the duration of the study. The results showed that 79% of individuals reported being satisfied with the smartphone-based EMA data collection method. We identified two attractors (fashion/style and features); three appropriation criteria (ease of use, intuitive user interface design, and simple instruction manual); three non-appropriation criteria (delaying the alarm, user interface elements, and burden of carrying the phone); and three reinforcers (the need for progress reports, the need for report on the relationship between pain and activity, and a simple user interface). This information is important in helping to understand the key factors that influence the use and adoption of the smartphone-based EMA data collection method and will assist in guiding its further development and implementation in low back pain studies.

The study had a number of limitations. It was a cross-sectional study with a modest sample size. Although there may be some selection bias toward those who were more health conscious, the findings are generalizable to individuals with low back pain in the community. We did not examine all aspects of the low back pain experience, such as the type and nature of the pain; however, we did collect data on three measures of pain intensity, including the current, maximum, and average pain, and obtained data in real time on a daily basis over two weeks and in the individual’s environment. Moreover, although we did not consider a wide range of measurements of functional activity, we did examine walking and sitting, which are the cornerstones of the assessment of function for individuals with low back pain. Although providing a formula based on the measures of pain and function, which indicates that the cohort or individual has improved, is beyond the scope of this study, this is an important area for future research. The study qualitatively used the MTA theoretical framework. The use of factor analysis in future research could provide additional evidence about the adoption and appropriateness of smartphone-based pain data collection.

## 6. Conclusions

This study showed that a smartphone-based EMA method allowing for the real-time, frequent collection of data in an individual’s natural environment could be used to capture pain and function data in a low back pain cohort. The work highlights the potential of the EMA method to enhance the collection of important, multidimensional outcome data in clinical trials of low back pain resulting in a more sensitive approach to identifying those with low back pain that are more likely to benefit from therapy. Moreover, it provides the opportunity to develop a personalized approach to the management of individuals with low back pain that may assist in reducing the burden of the condition.

## Figures and Tables

**Table 1:** Study protocol for smartphone-based EMA pain data collection method.

**Data Collection Device**	A secondary Android v8.1.0 smartphone (Nokia TA-1079) with no internet or cellular network connectivity with a belt pouch. Participants were given the smartphone with a pre-installed mobile app to collect pain data (pain intensity and function) at the start of the study. These smartphones were collected from users at the end of the study (week 2).
**Data Items** **Pain Intensity** **Function Activities**	Reporting period—Pain intensity was collected at a fixed time set by the user every day. The mobile data collection app asked the user: What is your current low back pain level from 0–10? What was your maximum low back pain level in the last 24 h from 0–10? What was your average low back pain in the last 24 h from 0—10? Reminder alarms were used in the mobile app to remind the user to enter their current pain levels at the time selected by the participant at the start of the study. Type of pain intensity—Numerical rating scale. Reporting period—Function data were captured automatically by the app running in the background continuously throughout the day. Type of sensor—Smartphone’s built-in accelerometer.
**Sampling Approach**	The fixed interval-based EMA method was used where participants were prompted once a day to enter their pain levels for 2 weeks. Function data were also captured continuously throughout the day for the 2-week period. Data were stored locally on the smartphone.
**Completion Rates** **Pain Intensity** **Function**	Completion rates were measured by the number of entries stored for pain intensity in the local database of the input device. Completion rates were determined by checking the local database of the smartphone for the timestamps of the accelerometer data recorded each day.
**Calculation of pain and functional measures**	Calculation of change in pain intensity, pain intensity index, walking index, and sitting index.

**Table 2:** Characteristics of study participants at baseline (n = 19).

Participant Characteristics	Values
Age (years), mean (SD)	47.6 (12.3)
Gender (female), n(%)	9 (47.4)
Body mass index (kg/m^2^), mean (SD)	27.9 (4.6)
Education (Bachelor’s degree or higher), n(%)	8 (42.1)
Health (Well or very well), n(%)	15 (78.9)
Employment (Full-time), n(%)	14 (73.3)
Employment Type (Office/professional), n(%)	15 (78.9)
Smoke (Currently non-smoker), n(%)	19 (100)

## Data Availability

Restrictions apply to the availability of these data since they contain health-related and sensitive data of the participants.

## Abbreviations

The following abbreviations are used in this manuscript:

EMA	Ecological Momentary Assessment
MTA	Model of Technology Appropriation
SUS	System Usability Scale
SD	Standard deviation
